# Stem Cells in Domestic Animals: Applications in Health and Production

**DOI:** 10.3390/ani12202753

**Published:** 2022-10-13

**Authors:** Eleonora Iacono, Barbara Merlo

**Affiliations:** 1 Department of Veterinary Medical Sciences, University of Bologna, Via Tolara di Sopra 50, Ozzano Emilia, 40064 Bologna, Italy; 2 Interdepartmental Centre for Industrial Research in Health Sciences and Technologies, University of Bologna, Via Tolara di Sopra 41/E, Ozzano Emilia, 40064 Bologna, Italy

In the last decade, researchers described Mesenchymal Stem/stromal cells (MSCs) as a possible population of cells for cell-based therapies in regenerative medicine, both for humans and animals.

The aim of this first article (and the aim of the next books in this collection) is to gather high-quality research and review articles that could broaden knowledge regarding the role of MSCs in domestic animals’ health and production.

Nowadays, in veterinary medicine, the owners require their animals to be treated with sophisticated and new treatments with the aim to improve the patient’s life quality but also, in the case of livestock animals, to improve the quality of products, aiming to preserve human health [1]. MSCs therapy could be then considered as an opportunity for researchers, veterinary practitioners, and animal owners for contributing to animal and human health and well-being.

Moreover, despite the fact that the mouse remains the proof-of-principle and allows to test a wide variety of therapeutic protocols, its homogeneous genetic background is not the same as that of humans, and the knockout model of pathology, experimentally induced, is not always a mirror of spontaneous pathology. In this context, domestic animals can be considered spontaneous models, both from a pathogenetic and therapeutic point of view, of hereditary and acquired pathologies. Moreover, especially regarding pets (i.e., dogs, cats), which share the same living environment as humans and are often subjected to the same stressful agents. For the reason listed above, domestic animals could be considered an important suitable model for human spontaneous diseases, as already stated in the guidelines emitted by the U.S. Food and Drug Administration (FDA) and by the European Medicines Agency (EMA). Regarding the role of domestic animals as in vivo models for human diseases, La Mantia et al. [2] in their systematic review reported the use of stem-cell treatment against acute or chronic ischemic cardiomyopathies in large animal models with regard to Left Ventricular Ejection Fraction (LVEF). The meta-analysis reported by the Authors showed that stem-cell therapy may improve heart function in large animal models and that the swine species is confirmed as a relevant animal model in the cardiovascular field. In this context, there is also the study of Garcia-Mendivil et al. [3] regarding the development of in vitro cellular models using ovine MSCs for prion neurodegenerative disorders affecting both humans and animals, particularly ruminants. Indeed, the response of ovine bone marrow-derived MSCs and their neuron-like derivatives to prion infection allowed us to find that BM-MSC-derived neuron-like cells could be a good candidate for developing in vitro studies.

As reported by Svoradova et al. [4], MSCs can be used as an avian culture model to better understand osteogenic, adipogenic, and myogenic pathways; moreover, chicken MSCs could also be used as a model for in vitro meat culture.

On the other hand, canine and equine species can be considered as both patients and clinical models. As reported by Prislin et al. [5] and Cequier et al. [6] in their reviews, canine and equine MSCs have been used for treating different pathologies, not only regarding the musculoskeletal system; but also involving ophthalmology, reproduction, gastroenterology, metabolic and neurologic disorders, and respiratory and integumentary systems.

To date, in canine and equine regenerative medicine, adult tissue, such as bone marrow (BM) and adipose tissue (AT) represent the most used sources of MSCs. Usually, these cells are cultured in a culture medium added with 10% of FBS (Foetal Bovine Serum). Despite that it is critically discussed for its ethical and healthy implications, FBS is still the gold standard for in vitro cultivation of MSCs. However, the trend in cell culture points to the use of xeno-free culture supplements, for which blood products, like platelet lysate (PL) from the same species, appear most promising. In PL the platelet-derived growth factors have already been released and cell membranes removed, thus it can be stored for a long time in the freezer. Moreover, positive and synergistic effects of PL might not only be achieved in cell culture but also in the subsequent therapeutic application when combined with MSCs. Hagen et al. [7], cultured canine and equine ATMSCs with 2.5% ad 10% of autologous PL. Cells cultured with 10% of FBS were used as control. It was found that PL did not support stem cell culture in dogs in the same beneficial way observed in the horse, revealing that using analogous canine and equine biologicals does not entail the same results. In fact, canine ATMSC cultured in medium supplemented with 2.5% and 10% of autologous PL changed their morphology, showed decreased metabolic activity, and increased apoptosis and necrosis; however, at passage five canine ATMSCs showed less genetic aberrations when cultured with 10% of PL than with FBS. It was concluded that, even if 10% of PL seems not lead to cell damage, considering the strong alteration observed in cell morphology and expansion, the use of PL cannot be recommended for canine ATMSC culture in its current form [7].

Due to invasive cell harvesting, donor site morbidity, cell amount, and characteristics related to donor age [8,9,10,11,12] connected to the use of BM and ATMSCs, in the last years researchers have directed their attention towards the study of new sources.

In canine regenerative medicine, an alternative to MSCs could be a stromal vascular fraction (SVF) non-cultured MSCs, separated from adipose tissue (AT). In recent clinical trials freshly isolated primary Stromal Vascular Fraction (SVF) cells have been used instead of cultured ATMSCs [13,14,15]. Hendawy et al. [16] demonstrated that in middle-aged and old dogs, the peri-ovarian harvesting site yielded higher SVF viability percentage, and viable cell number/gm fat than that of the other harvesting sites, such as subcutaneous abdominal fat and falciform ligament. In this study SVF cells from periovarian AT recorded revealed a higher expression of MSC markers (CD90, CD44, and CD29) compared to the other sites, with weak CD45 and CD34 expressions. Furthermore, the positive OCT-4 expression of SVF cells isolated from periovarian AT demonstrated their pluripotency, indicating them as a valid alternative to ATMSCs for cell therapy in canines. Similar data have been reported by Prislin et al. [5]. As reported by the Authors, canine SVF and ATMSCs treatments provide many benefits, in degenerative orthopedic pathologies, both in skin, bowel, and eye diseases [5].

Foetal fluids (amniotic fluid, umbilical cord blood), and foetal adnexa (Wharton’s jelly, amniotic membrane) have been identified as ideal alternative sources of MSCs in different animal species, such as horse [17,18,19], cattle [20,21], goat [22,23], and others. The benefits of these cells compared to adult MSCs are due to their origin from extraembryonic tissues; in fact, because they are at the maternal—foetal interface, these cells present low immunogenicity and immunomodulatory properties, making them a good candidate for allo- and xenotransplantation [24]. Iacono et al. [25], in their review, observed that, like reported in human and other animal species, also in dog MSCs derived from foetal fluid and adnexa may have an attraction compared to other established SCs in different clinical approaches, although more in vitro studies on their metabolism and clinical applications are needed to fully understand their properties and to establish the future clinical use in the treatment of various diseases. In this contest, Humenik et al. [26] described the effective protocols for the isolation of MSCs from canine bone marrow, adipose tissue, and amnion membrane, showing differences in yield of isolation, morphology, phenotype, multilineage potential, and proliferation activity.

While Humenik et al. compared canine MSCs isolated from AT, BM, and amniotic membrane, Merlo et al. compared equine ATMSCs and WJ (Wharton’s jelly) MSCs [26]. Due to the difficulties encountered by the practitioners in skin wound healing and the role of integrin in the reparative process, in this pilot study, the authors analyzed the effect of an  α4β1 integrin agonist on cell adhesion of equine AT and WJ-derived MSCs and investigated their adhesion ability to GM18 incorporated poly L-lactic acid (PLLA) scaffolds. The preliminary results reported in this paper represent a first step in the study of MSCs adhesion to PLLA scaffolds containing GM18, suggesting that WJ-MSCs might be more suitable than AT-MSCs. However, the results need to be confirmed by increasing the number of samples before drawing definite conclusions.

Additionally, the olfactory mucosa is a promising candidate for both humans and animals [27,28,29]. Mollichella et al. [30] evaluate the feasibility of collecting, purifying, and amplifying olphactory-ecto (OE) MSCs from the cat nasal cavity. The OEMSCs were isolated from biopsies and their stemness features as well as their mesodermal differentiation capabilities were characterized. This report shows for the first time that the isolation of OE-MSCs from cat olfactory mucosa is possible. These cells showed stemness features and multilineage differentiation capabilities, indicating they may be a promising tool for autologous grafts and feline regenerative medicine.

Beyond natural sources that are limited by stem cell availability, immune intolerance and lineage specification, bioengineered stem cells, such as induced pluripotent stem cells (IPSCs) have been developed [31]. In canine species, several reports have described the generation of IPSCs using retroviral or lentiviral transduction using Yamanaka’s factors [32,33,34]. Regarding viral reprogramming, different studies have shown that it can induce genomic integration and increase cell tumorigenic potential [35,36], so viral reprogramming is not suitable for clinical applications. In the study of Kim et al. [37], the 13-year-old canine fibroblasts were reprogrammed using a non-integrating Venezuelan equine encephalitis (VEE) RNA virus replicon, which has four reprogramming factors (collectively referred to as T7-VEE-OKS-iG and comprised of hOct4, hKlf4, hSox2, and hGlis1) and co-transfected with the T7-VEE-OKS-iG RNA and B18R mRNA. The derived colonies of putative canine IPSCs showed a resemblance to naïve iPSCs in their morphology (dome-shaped). The expression of endogenous pluripotency markers such as Oct4, Nanog, and Rex1 transcripts was confirmed, suggesting that induced cells were in the late intermediate stage of reprogramming. The reported research is the first of this type in canine species and, despite the good results obtained, it is a preliminary study and requires repeating with quantitative methodologies.

For therapeutic use, MSCs need to be isolated and expanded in vitro to obtain a sufficient amount for clinical application. Sometimes second or third applications could be needed, but long-term cultivation before therapeutic use is not recommended, since the cells may lose their stemness features and bacterial contamination may occur. For these reasons, it is very useful to cryopreserve these cells in order to gain a ready and controlled source of abundant autologous stem cells that maintain unaltered characteristics of the freshly isolated cells by preserving their vitality and maintaining their pluripotent phenotype. Di Bella et al. [38], evaluated the effects of 7-year-long cryopreservation using 10% DMSO and different FBS concentrations (from 10 to 90%). The Phenotype morphology, cell viability, differentiation, and proliferative potential, the expression of pluripotency markers in both fresh and thawed cells were analyzed. This study demonstrated that canine adipose tissue MSCs cryopreserved with more than 50% FBS and thawed after 7 years showed similar proliferative ability and morphological and molecular characteristics as fresh cells.

Usually, fresh or frozen-thawed cells after in vitro expansion in the laboratory are sent back to attending clinicians. As reported above, preserving MSCs characteristics en route from the laboratory to the clinic is fundamental for the success of the therapy. Due to the importance of this topic for veterinary regenerative medicine, in the last 10 years, different storage solutions, temperatures, and periods have been tested [39,40,41,42]. For equine MSCs isolated from AT and Wharton’s jelly (WJ), Iacono et al. [43] demonstrated that different types of MSCs react differently to the storage conditions frequently used for shipping them from the laboratory to the clinic. These conditions influence the viability and, depending on the cell type, they can also influence different MSCs characteristics. Particularly, equine WJMSCs need to be used quickly to maintain their viability. However, data recovered in vitro need to be compared with results obtained in vivo using cells shipped under tested conditions and with data obtained using frozen-thawed cells implanted directly.

Finally, among domestic animal species, camelids are an important source of both food and sport, as racing animals. In this case, they can present osteoarticular damages and the treatment with MSCs could be useful for accelerating the healing process. In this contest, Son et al. [44], for the first time, isolated, expanded, and studied cells isolated from BM and Synovial Fluid (SF) of Camelus dromedaries (camel). Due to the observed chondrogenic ability of SF-MSCs, they could be considered as a target cell source for future use in therapeutic cartilage regeneration in this species.

The contributors published in this first book collection, “Stem Cells in Domestic Animals: Applications in Health and Production,” are excellent examples of recent advances made in the field of stem/stromal cell research in veterinary medicine. We would like to thank the Authors for their excellent contributions and acknowledge Sandra Spatariu and the *Animals* Editorial Office for their support.

The Collection is open for submission of original manuscripts and reviews authored by outstanding experts in any aspect of stromal cell biology.
